# Utilization of multiple-criteria decision analysis (MCDA) to support healthcare decision-making FIFARMA, 2016

**DOI:** 10.1080/20016689.2017.1360545

**Published:** 2017-10-12

**Authors:** Julia I. Drake, Juan Carlos Trujillo de Hart, Clara Monleón, Walter Toro, Joice Valentim

**Affiliations:** ^a^ Genentech, Inc, San Francisco, CA, USA; ^b^ FIFARMA, Bogotá, D.C., Colombia; ^c^ JANSSEN, Venezuela, Central America and the Caribbean for JANSSEN, Panama; ^d^ AbbVie, Mettawa, IL, USA; ^e^ Roche Pharma, São Paulo, Brazil

**Keywords:** MCDA, HTA

## Abstract

**Background and objectives:**   MCDA is a decision-making tool with increasing use in the healthcare sector, including HTA (Health Technology Assessment). By applying multiple criteria, including innovation, in a comprehensive, structured and explicit manner, MCDA fosters a transparent, participative, consistent decision-making process taking into consideration values of all stakeholders.

This paper by FIFARMA (Latin American Federation of Pharmaceutical Industry) proposes the deliberative (partial) MCDA as a more pragmatic, agile approach, especially when newly implemented.

**Methods:** Literature review including real-world examples of effective MCDA implementation in healthcare decision making in both the public and private sector worldwide and in LA.

**Results and conclusion**: It is the view of FIFARMA that MCDA should strongly be considered as a tool to support HTA and broader healthcare decision making such as the contracts and tenders process in order to foster transparency, fairness, and collaboration amongst stakeholders.

## Introduction

This paper aims to present MCDA as a decision-making tool that can be applied in the healthcare sector due to the comprehensive and consistent yet flexible and transparent methodology, fostering collaboration amongst all healthcare stakeholders.

Current HTA approaches have overemphasized cost-effectiveness, incremental cost-effectiveness ratios (ICERs), and thresholds. Too much emphasis on cost-effectiveness presents limitations to holistic decision-making in that it excludes important factors such as innovation, disease severity, size of patient population, equity, or clinical guidelines [1]. Also, lack of cost-effectiveness is not a necessary or sufficient condition to reject access to treatments, especially in the case of rare diseases [2].

FIFARMA recognizes that while important, the role of cost-effectiveness is limited in helping choose among interventions that address a specific need. An effort should be made to allocate sufficient budget for the reimbursement of medicines, allowing more flexibility in healthcare decision-making. Systems that operate with a fixed cost-effectiveness threshold risk ignoring need: where society feels there is a great need, we are willing to pay much more and thus accept less efficiency, while in conditions perceived as minor or for which there are already very effective treatments we may be less willing to cover a new intervention, even if it has an excellent incremental cost-effectiveness ratio [3]. Emphasis on cost-effectiveness risks reducing equity in patient access to innovative medications. A 2014 study performed by the IMS Institute [3] compared cost-per QALY focused countries (CPQ) to countries that used a more holistic assessment approach (non-CPQ). This study concluded that:

- Patients in CPQ countries have less access to new cancer drugs than patients in non-CPQ countries, reimbursement decisions take longer, and new cancer drugs have historically been adopted more slowly at lower rates
- CPQ analyses are subject to many uncertainties and inconsistencies due to the nature of the variables used and their interpretation
- CPQ countries do not necessarily spend less overall on cancer, but they may achieve less for patients

Placing too much weight on a few criteria (efficacy and costs only) and a narrow perspective (not societal) can negatively impact patient equity in access to medications. FIFARMA recommends a multiple set of criteria for a more holistic and fair valuation approach.

The emphasis on and utilization of MCDA in healthcare decision-making has increased over the past 5 years, as demonstrated by the increase in publications since 2011 [1] and the prevalence of the topic in international healthcare congresses such as by the International Society of Pharmacoeconomic Outcomes Research (ISPOR), including the Latin America Conferences (e.g., the ISPOR 5^th^ Latin America Conference). ISPOR has also a dedicated Task Force for MCDA, *MCDA for Healthcare Decision Making, Emerging Good Practices Task Force* <http://www.ispor.org/Multi-Criteria-Decision-Analysis-guideline.asp> publishing reports and guidelines [4].

MCDA can be applied on a macro or micro level at various stages of the health technology development and assessment process. For this paper, emphasis will be placed on the utilization of MCDA in HTA processes across Latin America given decision-makers and appraisal committees can systematically appraise health technology in light of a multitude of decision criteria. It should be noted that MCDA can also be effectively used for tenders and contracts. FIFARMA and its represented member organizations maintain that healthcare decisions must be high-quality and autonomous, while relevant to the local market conditions and patient populations. A secondary goal of this paper is to show that the implementation of MCDA into healthcare decision-making is achievable by utilizing a systematic process, referencing real world examples and ongoing research from various decision-making bodies and countries. This paper is in line with FIFARMA’s position that an efficient HTA process should be transparent, fair, consultative, and focused on clinical excellence.

## MCDA as a deliberative tool in healthcare decision-making

A serious concern for patients, clinicians, and other stakeholders is the narrowness and lack of transparency in healthcare decision-making, especially in regard to coverage and reimbursement. Notable deficiencies in decision-making have prompted proposals to use MCDA because it has the potential to consider whatever criteria a stakeholder judges relevant [1]. MCDA takes into consideration the different institutional contexts while fostering a comprehensive, consistent, transparent, and flexible approach. By structuring the process of selection and evaluation of alternatives, MCDA quantifies evidence to identify best alternatives and helps eliminate contradictions between stakeholders [5]. An additional benefit is that MCDA can help sharpen signals to manufacturers in advance, to focus on providing data that matter most to decision-makers [6].

MCDA provides a framework for breaking down a complex decision into more manageable components, defining and understanding the relationship between these components. Additional, but not mandatory, steps would be measuring each component, and then combining them to identify solutions. MCDA also serves the difficult task of quantifying stakeholders´ priorities and preferences while forming a transparent link between judgments and decisions [7].

By taking into account and measuring criteria other than cost-effectiveness or budget impact, as for example equity in patient access and local health system priorities, MCDA ensures that social preferences, epidemiological priorities, and ethical values are not neglected in the decision-making process.

In regard to approaches within MCDA, FIFARMA supports the broad position approach adopted by the ISPOR Task Force [4]. The MCDA ISPOR TF included MCDA methods ‘that help deliberative discussions using explicitly defined criteria, but without quantitative modelling. … Decision makers can find this “partial” [deliberative] form of MCDA a useful way of summarizing the relevant evidence, to help structure their deliberations about which alternatives are best’ [4].

## MCDA criterion for inclusion

In order to facilitate a holistic and fair assessment of any healthcare technology, criteria included in the decision-making process must be relevant to local market conditions and comprehensive in that they include considerations of all relevant stakeholders and ethical values. The ISPOR MCDA Task Force recommends selecting and structuring criteria that are non-redundant and independent of the performance of other criteria [4].

It should be noted that while there is no rule on how many criteria should be included in an analysis, a higher number of criteria increases the complexity and cognitive effort, introducing the risk of tiring decision-makers and reducing the quality of responses [8]. It is FIFARMA’s position that criteria should remain straightforward in order to reduce the likelihood of uncertainty in outcomes.

Upon reviewing literature of MCDA utilized in healthcare decision-making [1,9–12] including the EVIDEM framework <https://www.evidem.org/>, FIFARMA recommends the criteria listed in Table 1. Additional criteria may be included if considered pertinent to the respective context (country, group of patients, indication etc.). A brief explanation of the criteria is provided in the footnotes. Selection and extensive definition and application of each criterion may be reached via consensus among all stakeholders respecting legitimacy of the participative process.

**Table 1.** FIFARMA recommended MCDA criteria for healthcare decision-making.

	Description of criteria
Quantitative criterion	**Added therapeutic benefit/innovation**^a^
	Improved efficacy/effectiveness
	Improved safety
	Unmet medical need addressed by new technology
	Quality of life (patients, families, caregivers)
	**Economic impact**^b^
	Economic impact from a societal perspective
	Local health system priorities
	Disease severity/progression^c^
	Health prioritization^d^
	Clinical guidelines and international health standards
	Completeness in international and local clinical practice guidelines
	Medications approved by globally recognized healthcare organizations^e^
	Quality of evidence
	Integrity and consistency of evidence
	Relevance and validity of evidence
**Qualitative criterion**	**Equity^f^**
	Patient access
	**Other**
	Sustainability of manufacturer business practices^g^
	Capacity of local system to use appropriate interventions

^a^Innovation (e.g., breakthrough designation therapy) can be captured via subcriteria (e.g., effectiveness, safety, QoL) or an as an independent criterion including broader definition (e.g., training and publications through clinical trials in country).

^b^Economic impact refers to net costs considering components such as lost productivity costs avoided (patients, families, caregivers) and improved efficiency in healthcare delivery.

^c^Disease severity/progression should consider survival prognosis with current standard of care, disease morbidity/clinical disability.

^d^Consideration of disease in regard to local system’s public health priorities.

^e^World Health Organization, Food & Drug Administration, European Medical Association.

^f^Equity means all patients have access to medications and treatment facilities regardless of income, gender, race, age, or any other status.

^g^Sustainability of manufacturer business practices refers to environmental aspects as well as consistency and reliability in the production of technologies.

Cost-effectiveness is not recommended as a criterion to avoid double-counting, given economic impact and effectiveness are already listed as separate criteria.

## Real-world examples

MCDA is more than an academic, theoretical decision-making model. It has been successfully applied to various therapeutic areas and types of healthcare decisions in countries around the world. Table 2 displays examples illustrating how decision-making bodies apply MCDA. This table is meant to capture actual utilization versus research or recommendations for utilization. Note that this is by no means an exhaustive list of real-world examples of MCDA utilization. Literature on the utilization of MCDA is sparse and it is assumed that many more formal and informal examples exist. The successful utilization of MCDA for various therapeutic areas, as cited, indicates that MCDA can be applied effectively to support healthcare decision-making.

**Table 2.** Real-world examples of MCDA utilization to support healthcare decision-making (ex-LatAm).

Country	Example(s) of utilization	Source
England/UK	i. Orphan drugs, AGNSS/NICE ii. Respiratory, mental, children’s health, cardiovascular, and cancer interventions, NHS/Primary Care Trusts iii. Major capital expenditures, NHS	Devlin & Sussex [13] Adams et al. [14] Airoldi et al. [15]
USA	i. Diagnosis and treatment decisions ii. Clinical trial design	Adunlin et al. [16] Guest et al. [17]
Canada	i. Healthcare priority-setting ii. Budgeting iii. Interventions for chronic non-cancer pain	Diaby et al. [18] Tony et al. [19]
Germany	Incorporation of patient involvement with MCDA quantitative approaches, IQWIG	Danner et al. [20]
Sweden	i. Orphan drug coverage, TLV ii. High-cost biologics, TLV	World Health Organization [21] Deans et al. [22]
Denmark	Orphan drug coverage	Deans et al. [22]
Finland	Obesity research and prevention	Borg & Fogelhol [23]
The Netherlands	i. Orphan drug coverage ii. Publicly funded healthcare priority-setting iii. Ankle-foot repair in stroke	Van Til [24] Devlin & Sussex [13] Baeten et al. [25]
Italy	EVIDEM framework used with medical devices, diagnostic assessments, and pharmaceuticals	Radaelli et al. [26]
France	Screenings	World Health Organization [21]
Norway	Healthcare priority-setting	Defechereux et al. [27]
Hungary	Hospital medical technologies, OEP	Devlin et al. [4]
Scotland	Orphan drug coverage, NHS	Kanters et al. [28]
New Zealand	Algorithmic approach using 1000Minds software used to analyze coronary artery bypass graft surgery, MoH	Devlin & Sussex [13] Hansen et al. [29]
South Africa	Private health plan used for liquid-based cytology for cervical cancer screening	Miot et al. [30]
Ghana	Healthcare priority-setting	Jehu-Appiah [31]
Thailand	Health interventions in the universal health coverage benefit package, NHS	Youngkong et al. [32]
Israel	New healthcare technologies, Health Basket Committee	Devlin & Sussex [13]

MCDA is also being considered in many markets across LatAm, as reflected in Table 3. It should be noted that utilization of MCDA in LatAm is even more sparsely published. Information has been gleaned from local market insight as well as the 2015 ISPOR 5^th^ Latin America Conference presentations, with the exception of the literature cited in the table.

**Table 3.** Examples of recommended or actual real-world utilization of MCDA in LATAM.

Country	Implementation progress by stakeholders	Source
Brazil	a. MCDA proposal for rare disease, Interfarma b. MCDA used for hospital investment, RJ Uni. Hospital	Brito et al. [33] Nobre et al. [34]
Argentina	Incorporation of MCDA into the SUMAR Project, Ministry of Health	Pichon-Riviere [35]
Colombia	Pilot completed in 2013 and MCDA implemented for healthcare prioritization, IETS	Jaramillo [36]
Chile	Utilization of MCDA in considering tender offers, University of Chile Hospital	‘Informe de Evaluacíon’ [37]
Dominican Republic	Seeking insight from external consultants, Ministry of Public Health	Espinoza [38]
Ecuador	Prioritization process for HTA utilizing MCDA recommended, Ministry of Public Health	Sotomayer et al. [39]

The purpose of illustrating these real-world applications of MCDA is to stress that MCDA can be implemented as a useful tool to support healthcare decision-making and foster a fair and transparent decision-making process with a patient-centric approach. It is the position of FIFARMA that MCDA can be broadly applied to the HTA process in order to support healthcare decision-making.

## Implementation considerations

In considering the implementation of MCDA or any other healthcare decision-making process, sufficient budget should first be allocated for the reimbursement of medicines. Furthermore, policy-setting should be pro-innovation, meaning decision-makers value additional clinical benefits and unmet medical needs achieved by new healthcare technologies.

The main aspects of any MCDA method are (1) the alternatives to be appraised and (2) the criteria against which the alternatives are appraised. Additional steps for quantitative or complete MCDA would still require (3) scores that reflect the value of an alternative’s expected performance on the criteria and (4) criteria weights that measure the relative importance of each criterion as compared with others [5]. Key steps to conducting an MCDA analysis as adapted from the ISPOR MCDA Task Force [4] and MCDM (Multiple Criteria Decision Making) Tool (ZRx Outcomes Resources Inc.) are reflected in Table 4. FIFARMA recommends the first three steps, which constitute the deliberative, partial MCDA.

**Table 4.** MCDA implementation considerations, Deliberative MCDA highlighted.

Steps to implementation	Description	
**Define the objectives**	**Identify type of decision, alternatives, and relevant stakeholders**	
**Select the criteria**	**Influenced by scientific literature and specific local needs**	**Deliberative MCDA**
**Measure the alternative’s performance**	**Options must be able to incorporate qualitative and quantitative information, ‘performance matrix’ to summarize**	
Score options and aggregate scores	Scoring helps produce an overall estimate of value pay-off for each alternative	
Apply scores and weights to rank alternatives	Multiply the alternatives’ scores on the criteria by the weights and sum to get the total scores	
Explore and analyze uncertainty	Perform a scenario or sensitivity analysis	
Validate and interpret finds	Interpret outputs and align with decision-maker priorities to support decision-making	

Typically, the most complex part of the MCDA process is determining how to measure a criterion’s performance and manage uncertainty in outcomes.^1^

It is out of the scope of this paper to do a deep dive into measurement models. FIFARMA’s recommendation is that MCDA be implemented in a deliberative manner and not by a rigid, fixed mechanism.

As with any complex decision-making process, the output of MCDA is subject to uncertainty and the impact of this uncertainty should be addressed. It is the view of the ISPOR MCDA Task Force and FIFARMA that uncertainty not be included as a criterion in MCDA. A scenario analysis or sensitivity analysis is recommended for considering this impact, but it is out of the scope of this paper to analyze and explain these approaches [8].

Although MCDA may present such methodological variety, its main contribution is indeed the deliberative process still allowed by the partial approach. MCDA has proven significant value in that it is possible to systematically assess any disease in the context of the treatment that is available and local market priorities [40]. Consensus, a formalized approach, and validation of the process are required for MCDA implementation. Organizational change will also be necessary and therefore engaging experienced independent consultants is recommended.

MCDA can be implemented at the macro and micro levels, such as national and state, or at the hospital and healthcare-provider levels. It should be noted that successful implementation of MCDA will be an iterative process. Prior to a broad roll-out of MCDA, it is recommended to pilot the methodology, in prioritized high-cost disease states such as oncology or orphan diseases, for which cost-effectiveness limitations are even stronger.

## Summary

In conclusion, MCDA is a structured, transparent, participative, consistent, and legitimate tool to support healthcare decision-making as it provides a systematic framework for breaking down a complex decision into a transparent and rational process that incorporates the priorities and values of stakeholders. Real-world examples of effective MCDA implementation in healthcare decision-making in both the public and private sector confirm that MCDA can be applied to facilitate holistic assessments. It is the view of FIFARMA that MCDA should strongly be considered as a tool to support HTA and broader healthcare decision-making such as the contracts and tenders process in order to foster transparency, fairness, and collaboration amongst stakeholders.

## Appendix

- REAL-WORLD EXAMPLES OF MCDA UTILIZATION
- The UK
- The Advisory Group for National Specialized Services (AGNSS) developed a framework utilizing MCDA to support reimbursement decisions for orphan drugs. In 2012, the National Institute for Health and Care Excellence (NICE) then assumed responsibility for analyzing orphan drugs using the framework presented by AGNSS.
- Use of MCDA in a Local Healthcare Plan in the English NHS: MCDA was utilized to support the Isle of Wight Primary Care Trust (PCT) in the allocation of resources across 21 interventions in five priority areas: respiratory, mental, and children’s health, cardiovascular disease, and cancer. Interventions were assessed on three criteria: increased health (reduced mortality and increased quality of life); reduced health inequalities; and operational and political feasibility. The resulting estimate of value was combined with data on the cost to estimate ‘value-cost triangles’, which were ordered to construct an efficiency frontier.

Key stakeholders were engaged in the analysis: clinicians, council representatives, voluntary sector representatives, nurses, public and patients’ representatives, hospital managers, and the ambulance service. Participants agreed on the interventions to be evaluated and the research team collected data on the performance of these interventions. Stakeholders scored the interventions using a 0–100 visual analogue scale, and weighted the criteria using a swing weighting approach.

Interviews with participants revealed the benefit of the MCDA approach. First, most stakeholders found the approach accessible, something the authors attribute to their being continuously engaged in the design and implementation of the MCDA, and the use of visual aids to communicate results. Second, stakeholders found the approach acceptable, except in a minority of cases, such as palliative care, which generated benefits that fell beyond the three criteria. Third, stakeholders appreciated the logic of the approach, which they considered ‘an advance on just sitting around a table and talking it through’ [15].

- Canada

Tramadol for chronic non-cancer pain was selected by the public health plan for assessment. Based on extensive literature review 14 criteria for the MCDA Core Model and six qualitative criteria for the Contextual Tool as developed by EVIDEM were utilized. During workshop sessions, committee members tested the framework in three steps by assigning: (1) weights to each criterion of the MCDA Core Model representing individual perspective; (2) scores for tramadol for each criterion of the MCDA Core Model; and (3) qualitative impacts of criteria of the Contextual Tool on the appraisal. Utility and reliability of the approach were explored through discussion, survey, and test-retest. Agreement between test and retest data was analyzed by calculating intra-rater correlation coefficients (ICCs) for weights, scores and MCDA value estimates…. Overall, the framework was found useful by the drug advisory committee in supporting systematic consideration of a broad range of criteria to promote a consistent approach to appraising healthcare interventions [19].

(B) Germany: the case of IQWIG

In 2010, the German Institute for Quality and Efficiency in Healthcare (IQWiG) initiated a study to explore the use of MCDA methods as a means of incorporating patient involvement into its HTA process. Patient involvement is widely acknowledged to be important in HTA and healthcare decision-making. However, quantitative approaches to ascertain patients’ preferences for treatment endpoints are not yet established. The project used the analytic hierarchy process (AHP) and conjoint analysis (CA) as preference-elicitation methods for use in HTA.

The AHP study included two AHP workshops: one with 12 patients and one with seven healthcare professionals. In the workshops, patients and professionals rated their preferences with respect to the importance of different endpoints of antidepressant treatment by a pairwise comparison of individual endpoints. These comparisons were performed and evaluated by the AHP method and relative weights were generated for each endpoint.

A discrete-choice experiment (DCE), the choice-based variation of CA, was used. Patients and healthcare professionals were asked to choose between two (fictitious) hepatitis C treatment alternatives that were composed of various treatment characteristics (attributes, e.g., outcomes) and that differed according to the levels of the characteristics. The results of all of these choices were analyzed using logistic regression models to estimate the importance (weighting) of the individual treatment attributes. Overall, MCDA was carried out in a real-world context and was successfully used to increase rational, transparent, and fair priority-setting [20].

(C) Lombardy, Italy

This study describes the health technology assessment (HTA) framework introduced by Regione Lombardia to regulate the introduction of new technologies. The study outlines the process and dimensions adopted to prioritize, assess, and appraise the requests of new technologies.

The HTA framework incorporates and adapts elements from the EUnetHTA Core Model and the EVIDEM framework. It includes dimensions, topics, and issues provided by the EUnetHTA Core Model to collect data and process the assessment. However, decision-making is supported by the criteria and Multi-Criteria Decision Analysis technique from the EVIDEM consortium.

The HTA framework moves along three process stages: (i) prioritization of requests, (ii) assessment of prioritized technology, and (iii) appraisal of technology in support of decision-making. Requests received by Regione Lombardia are first prioritized according to their relevance along eight dimensions (e.g., costs, efficiency and efficacy, organizational impact, safety). Evidence about the impacts of the prioritized technologies is then collected following the issues and topics provided by the EUnetHTA Core Model. Finally, the Multi-Criteria Decision Analysis technique is used to appraise the novel technology and support Regione Lombardia decision-making.

The VTS (Valutazione delle Tecnologie Sanitarie) framework was successfully implemented at the end of 2011. From its inception, 26 technologies have been processed [26].

(D) Hungary

MCDA was introduced in Hungary in 2010 for the evaluation of new hospital medical technologies. The MDCA includes the evaluation of six criteria: healthcare priorities, severity of disease, equity, cost-effectiveness and quality of life, budget impact, and international reputation. These criteria and their weights were established by a committee comprising the healthcare financing agency, the Ministry of Health, clinical experts, and health economists. Weights were determined by allocating 100 points across the criteria to reflect their relative importance. The criteria and weights were submitted to other stakeholders for validation. Manufacturers submit a formal HTA report, including a health-economic analysis, clinical evaluation, clinical expert opinion, and detailed cost calculation. Technologies are then scored against the criteria by the healthcare financing agency. A technology is considered suitable for reimbursement if it achieves 60% of total available points, and achieves at least 40% of the available points on all the six criteria. The points achieved by a technology are not made public. Between 2010 and 2013, 14 applications were consideration using the MCDA method. Six resulted in a formal decision (supporting or rejecting). Three were terminated because of a lack of information. Five cases are still in progress [4].

(E) South Africa

MCDA was utilized to assess liquid-based cytology for cervical cancer screening for a private health plan. The committee utilized 14 criteria input into the MCDA model and four contextual criterions, extracted from a literature review and input from the health plan. A workshop was held in which the 14 criteria were weighted and scored and the impacts of the four contextual criteria were discussed. When appraising LBC for cervical cancer screening, the committee assigned the highest scores to ‘Relevance and validity of evidence’ and ‘Disease severity’. Overall, the committee felt the framework brought greater clarity to the decision-making process and was easily adaptable to different types of health interventions. The EVIDEM framework was easily adapted to evaluating a screening technology in South Africa, thereby broadening its applicability in healthcare decision-making [30].

(F) Thailand

MCDA was successfully utilized for including health interventions in the universal health coverage benefit package in Thailand. In 2012, the National Health Security Office, the institute managing the Universal Coverage Scheme in Thailand, called for more rational, transparent, and fair decisions on the public reimbursement of health interventions. To address this issue, ‘MCDA was applied in four steps: 1) 17 interventions were nominated for assessment; 2) nine interventions were selected for further quantitative assessment on the basis of the following criteria: size of population affected by disease, severity of disease, effectiveness of health intervention, variation in practice, economic impact on household expenditure, and equity and social implications; 3) these interventions were then assessed in terms of cost-effectiveness and budget impact; and 4) decision makers qualitatively appraised, deliberated, and reached consensus on which interventions should be adopted in the package’ [32].

(G) New Zealand: 1000Minds tool utilization

The MCDA process supported by internet‐based software 1000Minds was performed by a working group of clinical leaders for the elective service concerned, in consultation with patient groups and other clinicians. The MCDA process consisted of seven steps, as below

(H) Rank patient case vignettes using individual clinical judgments and then by consensus.
(I) Draft the criteria and the categories within each criterion for prioritizing patients.
(J) Pre‐test the criteria and categories and refine them.
(K) Consult with patient groups and other clinicians.
(L) Determine the point values for the criteria and categories.
(M) Check the test‐retest reliability and face validity of the points system.
(N) Revise the points system as new evidence emerges or clinical judgments change.

A survey of the participating clinicians revealed high levels of ‘user’ satisfaction with the method/software. The CABG points systems have been formally accepted and are in use throughout NZ. NZ’s Ministry of Health has led projects to create and validate new points systems for elective services – with the ultimate goal of more equitable access and better patient outcomes overall. Inspired by NZ’s success, since 2008 the same process has been used in the public health systems of Canada’s western provinces [29].

A. OVERVIEW OF MCDA PROCESS & COMPARISON OF APPROACHES

Classification of MCDA Methods

AHP: analytical hierarchy process

PBMA: program budgeting and marginal analysis

ELECTRE: elimination and choice expressing reality

PROMETHEE-GAIA: preference ranking organization method for enrichment evaluations

Comparison of different MCDA models

**Table:**

	Value measurement models	Outranking approach	Goal programming
Weights	Swing weights are used to capture both the effect of measurement scales and the importance of the criteria Weights should satisfy preferential independence of criteria and the trade-off requirements	Weights are uninfluenced by the scale of the value functions. They convey the relative importance of criteria in the assertion that one alternative is better than the other Weights do not have to satisfy any condition	Weights are attached to the deviations and represent the relative importance of criteria by specifying an overall measure of deviation from the goals Weights do not have to satisfy any conditions
Measuring the performance of the criteria	Performance scores v_i_(a), monotonic functions of the attribute values z_i_(a), need to be developed for all criteria i. Significant effort is needed to develop performance scores	Intuitive and easy to follow. With the right software, assumptions can be changed and results can be observed almost instantaneously	Easy to understand but requires significant computational time to provide results. Real-time updating is not possible
Presentation of the results	Easy to follow and enables further deliberation, well suited for good visual presentation of the results	Moderately easy to follow, can be presented visually but difficult with multiple alternatives	Results easy to follow, but they cannot be represented visually
Incorporating uncertainty	Probabilistic sensitivity analysis can be used to propagate parameter uncertainty quite easily	Moderately difficult to include uncertainty, need specialist software	Quite difficult to include uncertainty, complex stochastic programming techniques needed

Source: Thokala and Duenas [5].

B. STRENGTHS & CHALLENGES OF MCDA

**Table:**

Strengths/opportunities	Challenges
Utility	
Transparency, if algorithms are public Transferability, adaptable to local markets Flexibility, can vary by therapeutic area Consistent/systematic decision progress Identifies social values and encourages unbiased decision-making Incorporates societal preferences	Perception of complexity in implementation Integration into existing processes Risk of using MCDA as a formula rather than as support for decision-making/priority-setting Roles of decision-makers in making scientific and social value judgments Requires significant resources to capture population preferences
Methodology	
Inclusion of innovation as a criterion More holistic, considering all relevant dimensions, not only economic dimensions Pragmatic, user-oriented, and modular Detailed instructions	Criteria selection and measurement MCDA model selection and mathematics Developing a consistent framework to represent the relative importance of each criterion to society Managing uncertainty in meaning of results
Data requirements	
Comprehensive but modular Leverages technology	Data synthesis by criteria Web integration
Capacity/training requirements	
Community of users and developers Open participation to all stakeholders, likely via representatives from societies	New paradigm, limited training and understanding Limited MCDA expertise in healthcare

Source: Adapted from ‘Lessons learned from a multi-criteria decision analysis [MCDA] framework’ EVIDEM presentation to the Institute of Medicine in Washington DC, July 2011, and Mondher Toumi [12].

^1^The types of MCDA models that are most commonly used include weighted sum or value measurement, outranking, and goals programming. Although there is no consensus on the best MCDA to utilize, the weighted sum or value measurement model is most utilized in healthcare decision-making [1]. The value measurement model assesses interventions based on an overall benefit score. This benefit score is calculated as the weighted average of the criteria.

**Table:**

Key points for decision-makers
*MCDA is a decision-making tool with increasing use in the healthcare sector, including HTA (Health Technology Assessment). By using multiple criteria in a comprehensive, structured, and explicit manner, MCDA fosters a transparent, participative, consistent, and legitimate decision-making process. A deliberative^a^ (partial) MCDA may be a more pragmatic, agile approach, especially when newly implemented.*

^a^A deliberative MCDA can also be considered as Multiple Criteria Decision-Making (MCDM].
